# Interacting effects of age, density, and weather on survival and current reproduction for a large mammal

**DOI:** 10.1002/ece3.1250

**Published:** 2014-09-18

**Authors:** Emmanuelle Richard, Steven E Simpson, Sarah A Medill, Philip D McLoughlin

**Affiliations:** 1 Department of Biology, University of Saskatchewan112 Science Place, Saskatoon, SK, S7N 5E2, Canada

**Keywords:** Density dependence, density independence, feral horses, life history theory, reproduction, survival

## Abstract

Individual-based study of natural populations allows for accurate and precise estimation of fitness components and the extent to which they might vary with ecological conditions. By tracking the fates of all 701 horses known to have lived on Sable Island, Canada, from 2009 to 2013 (where there is no predation, human interference, or interspecific competition for food), we present a detailed analysis of structured population dynamics with focus on interacting effects of intraspecific competition and weather on reproduction and survival. Annual survival of adult females (0.866 ± 0.107 [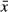 ± SE]) was lower than that of 3-year-olds (0.955 ± 0.051), although annual fecundity (producing a foal in a year that was observed during our census) was higher in adults (0.616 ± 0.023) compared to 3-year-olds (0.402 ± 0.054). Milder winters and lower densities during gestation increased fecundity. Density negatively impacted survival for all age and sex categories; however, highest adult female survival was observed during high-density years coupled with a harsh winter, the result expected if pregnancy loss during winter or loss of foals in spring improved survival. Three-year-old females, which reproduced at lower rates, experienced higher survival than adults. Our results contrast with a previous study of feral horses that suggested recently feral ungulates might be artificially selected to reproduce even when costs to survival are high. In part, this may be because of the comparably long history of feralization (250 years; at least 25 generations) for Sable Island horses.

## Introduction

The relationship between traits of life history and the environment, with subsequent effects on population dynamics, is often complex and may include interactions between both density-dependent and density-independent processes. For example, Portier et al. (1998) showed that for juvenile bighorn sheep (*Ovis canadensis*), effects of weather on lamb survival were in some cases mediated by an interaction with population density (spring and winter temperatures had a positive effect on neonatal survival only when population density was high). Golden eagles (*Aquila chrysaetos*) hatch earlier when prey is abundant, but this is delayed after severe winters (Steenhof et al. 1997). There is a negative interaction between mean summer temperature and density for Chinook salmon (*Oncorhynchus tshawytscha*), with growth in body size positively correlating with temperature at low density, but vice versa at high density (Crozier et al. 2010). Further, effects of these interactions may depend on organism state. In bighorn sheep and Soay sheep (*Ovis aries*), interacting effects of density and weather can vary for animals of different ages and sexes, leading at times to different population dynamics under the same weather conditions depending on population age and sex structure (Festa-Bianchet et al. 1998; Coulson et al. 2001).

More recently, investigations into how density-dependent and independent processes might interact has factored into research on eco-evolutionary dynamics (Schoener 2011). For example, Ozgul et al. (2010) showed how rapid environmental change can affect timing of hibernation in yellow-bellied marmots (*Marmota flaviventris*), leading to longer growing seasons, larger body masses, and subsequent effects on demography including a shift in the onset of density dependence. How density-dependent and density-independent processes interact also has important implications for life history theory. For example, the trade-off between survival and current reproduction is hypothesized to appear under food stress (Stearns 1992) and thus may vary in response to interacting effects of factors such as conspecific density and modifying effects of weather.

In searching for ecological mechanisms, the most useful approach to understanding population dynamics is reductionist (Turchin 2003; Clutton-Brock and Sheldon 2010), whereby the basis of population dynamics is studied by the properties of individuals. This may include properties of age and sex; behavior and physiology; individual consumption, growth, and reproduction; individual movements and habitat use; and interactions with other species and the abiotic environment. Comprehensive, individual-based studies of this nature remain rare, however – especially for large, long-lived animals (Clutton-Brock and Sheldon 2010). Fewer yet are long-term studies where ecological interactions are simplified enough to isolate main effects (e.g., interspecific competition from intraspec0ific competition) or where there is no human interference in a population's natural dynamics.

**Figure 1:**
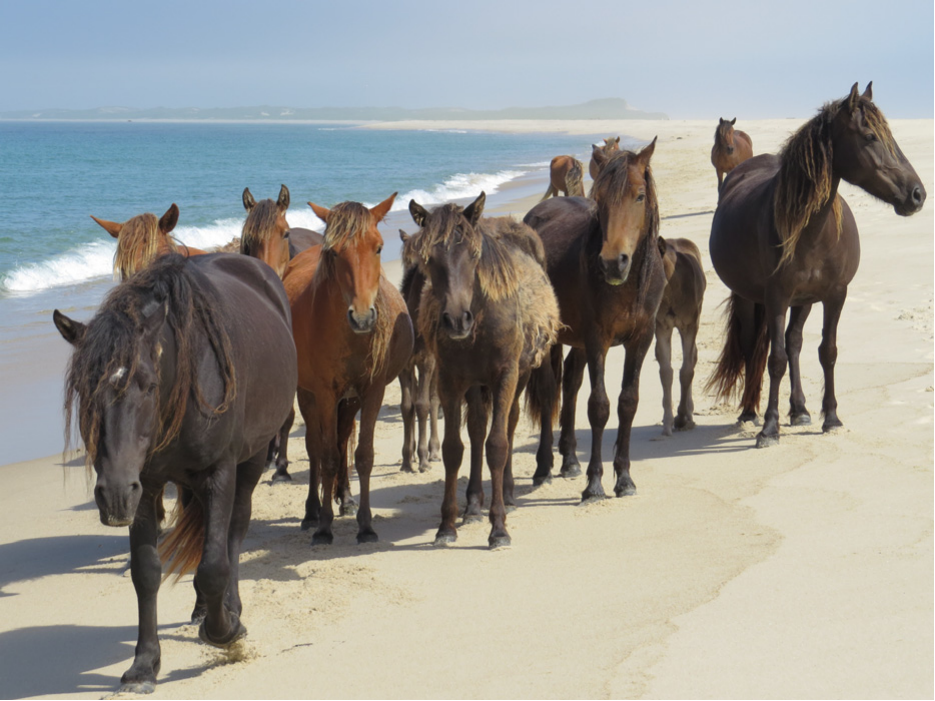
A band of feral horses on Sable Island, Nova Scotia, Canada. Photo © Philip D. McLoughlin.

The isolated and protected population of feral horses on Sable Island, Nova Scotia, Canada (Fig.1) is free from predation, human interference, and competition with other species (the horses are the only terrestrial mammal). These circumstances allowed us to conduct a focused study of the interacting effects of intraspecific competition (conspecific density) and weather on the dynamics of a large mammal. Here, we present a detailed, individual-based population analysis spanning years 2009–2013 (including life histories for all 701 horses known to be alive during the period). Our study included the following: (1) a comparative survival analysis for females (transition matrices); (2) an age- and sex-structured analysis of survival with density dependence; and (3) a comparative analysis of interacting effects of local weather and density on reproducing females (3-year-olds and adults [aged 4+]). We expected both density- and age-related effects on survival, as previously reported for feral horses during a period of population growth (review in Grange et al. 2009), including negative density effects on survival for all age groups, and lower juvenile compared to ≥2-year-old survival rates, with highest survival in females close to first age at parturition, which may obtain energy at adult rates but not experience costs of reproduction. We expected that severe winters, by impacting maternal nutrition during late gestation, may lead to lower *in utero* and preweaning foal survival (Adams et al. 1995). We expected males and females to survive at similar rates (Grange et al. 2009). We predicted that elasticity of adult survival would be higher than that of other vital rates, as shown in previous demographic analyses on large herbivores (for instance Heppell et al. 2000; Nelson and Peek 1982). Finally, following Grange et al. (2009), we predicted high fecundity in adult females irrespective of environmental conditions (density and weather), as a result of artificial selection for high reproductive performance, which is known to have occurred in other domesticated ungulates including horses.

## Materials and Method

### Study area

Sable Island National Park Reserve (43°55′ N; 60°00′ W) is a crescent-shaped, sand island located 275 km southeast of Halifax, Nova Scotia, Canada. It is nearly 50 km long and 1.25 km at its widest. The grassland-dominated, treeless island supports a free-ranging population of horses, which has largely been feral since their introduction in the mid-1700s; they remain as the island's only terrestrial mammal (Christie 1995). Details of the study area and population are presented in Contasti et al. (2012) and Van Beest et al. (2014).

### Data collection

We performed population censuses and collected field observations for the entire horse population (*N *=* *458, 507, 461, 538, and 559, respectively, from 2009 to 2013 (Table1, densities include observed individuals plus known births postcensus, determined the following year). Individuals were identified using digital photographs, and at each observation, we recorded sex, field age, and reproductive status. We used high-resolution aerial photography from January 2010 to confirm that our censuses accounted for >99% of the horses found on the island; therefore, any horse which we failed to find the following year was considered dead. We aged horses based on known year of birth (for horses identified in our first years of data collection as yearlings in 2007 [foals in 2006]), or based on size and appearance (using as a guide the appearances of our known-aged horses) if birth year was not known. For analysis, we used age categories: foal (age 0), yearling (age 1), 2-year-olds, 3-year-olds, and adult (ages 4+). To further improve our accuracy of aging subadult horses, we initiated our matrices as starting in 2009 and did not take into account our initial surveys in 2007 and 2008.

**Table 1:** Numbers of males and female horses observed on Sable Island, Canada, from 2009 to 2013. Total number of individual life histories in the sample is 701 horses

Year	Foals	Yearling	2-year-olds	3-year-olds	Adults
Females					
2009	31	34	18	22	106
2010	47	31	29	17	119
2011	36	33	23	20	105
2012	54	33	30	23	123
2013	34	48	30	26	122
Males					
2009	49	37	25	22	114
2010	40	43	35	25	121
2011	23	30	39	27	125
2012	42	19	28	39	147
2013	46	35	18	25	175

We obtained climate data from a historical database on weather for Sable Island's meteorological station. First, we obtained the average of the minimum temperature in January that we believed to impact on female body condition on Sable Island (S.E. Simpson, unpubl. results). Access to vegetation has an impact on demography of horses (Contasti et al. 2013). As in Gaillard et al. (2013), we used the number of degree-days higher than 7°C from February to April as a measure of spring phenology because vegetation flush occurs increasingly earlier as the number of degree-days >7°C increases (Wilson and Barnett 1983). A critical temperature for young horses is 0°C (Cymbaluk 1990), and so, we also identified for each year of study the number of days below zero during winter.

### Statistical analyses

**Figure 2:**
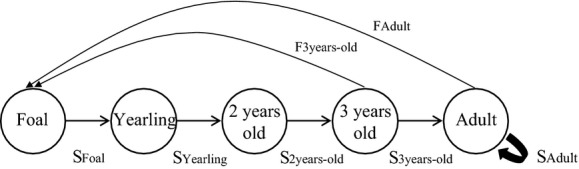
Life cycle graph of female Sable Island feral horses. Foal (age 0), yearling (age 1, prebreeder), 2-year-olds (may mate for the first time), 3-year-olds (females may foal for the first time), adult (females of prime age). All *S*_*i*_ are age-specific survival probabilities and *F*_*i*_ are age-specific fertilities (following Caswell 2001). Transition between ages occurs once per year.

Horse surveys on Sable Island allowed us to obtain a time series of near perfect estimates of demographic parameters from 2009 to 2013; as we were able to identify each horse on the island, we do not account for sampling error in our analyses. Our life cycle graph for Sable Island horses (Fig.2) included five age classes and seven demographic parameters. The data we used to calculate survival and reproduction were collected near the end of the seasonal birth pulse (July–end August). We coded our demographic data as binary variables: “0” for “dead” or “no reproduction” and “1” for “alive” or “reproduction” during the biological year (for survival and reproduction [fecundity], respectively). Here, fecundity corresponds to the probability of giving birth to a full-term foal (because horses are monotocous) and having that foal survive until the end of the next year's annual census. Hence, fecundity includes the loss of some foals as newborns prior to sampling, at birth, or *in utero,* but not overwinter survival to yearling age. Annual survival was age-specific and was calculated from 1 April of the year (*t*) to 31 March of each (*t *+* *1) from 701 individual life histories (monitored between 2009 and 2013; Table1). A cohort is defined as all individuals born in the same biological year. To account for the relatively long birthing period when assessing foal survival, foals born between November (*t*) and 31 March (*t +* 1) are included in the cohort of year (*t*) (following Grange et al. 2009). We defined survival of age-class *i* (*S*_*i*_) as the number of surviving individuals at *t *+* *1. For example, foal survival was calculated as the ratio between the number of yearlings at *t *+* *1 and the number of foals at *t*. We distinguish the average number of offspring produced per female of age-class *i* at time *t* from the average number of offspring per individual of age *i* at time *t* that are counted at time *t *+* *1, that is, following a postbirth pulse census. This last estimate is used in Leslie matrices and by convention (based largely on its application to human demography) is termed “fertility” or *F*_*i*_ (Caswell 2001).

We constructed a female-based age-structured projection (Leslie) matrix, for each interval (2009–2010, 2010–2011, 2011–2012, and 2012–2013) to describe current population dynamics using standard matrix calculations (Caswell 2001). From this, we estimated elasticity of rates (the proportional change in the finite rate of population growth, *λ*, when changing a given parameter by 1%) following Caswell (2001). Elasticity values estimate the influence that a potential change in an age-specific vital rate has on *λ*. For example, higher ranked elasticity values for adult versus juvenile survival in mammals (e.g., Brault and Caswell 1993 for orca *Orcinus orca*; Gaillard et al. 2000 for a review of ungulates) or birds (see Sæther and Bakke 2000 for a review) suggest that a proportional change in the former would have a stronger influence on population trajectory than the latter. We used our four intervals to project the average matrix across 20 years in order to determine trajectory of population growth (*λ*) and compare the age structure of our population to that of the stable age distribution.

**Figure 3:**
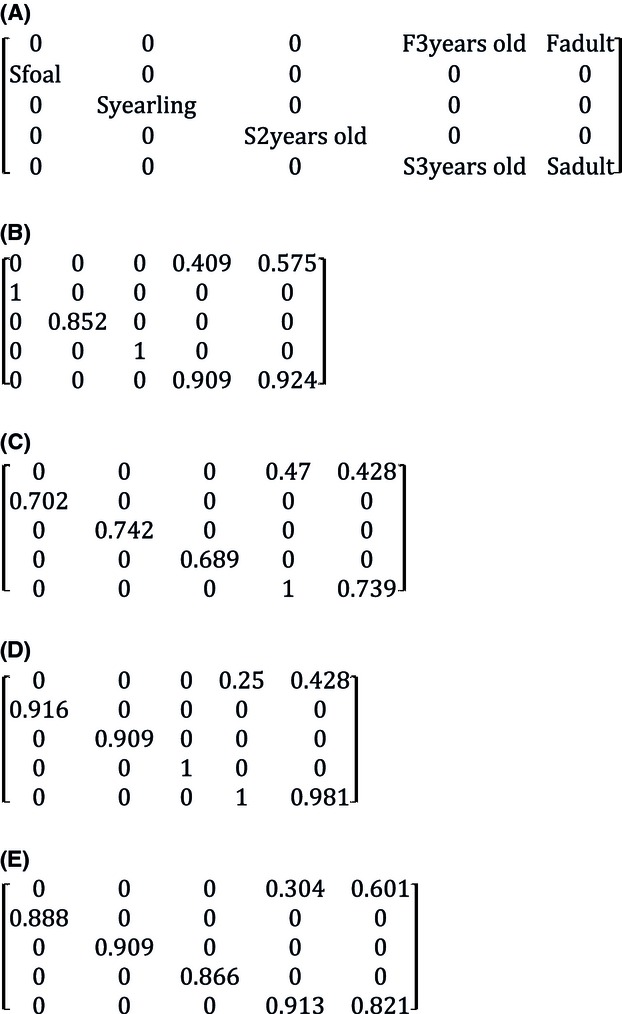
Female-based age-structured transition matrices for Sable Island horses. (A) General matrix structure indicates locations of survival and fertility rates for each year. Notations are: foal survival (*S*_foal_), yearling survival (*S*_yearling_), 2-year-old survival (*S*_2yearsold_), 3-year-old survival (*S*_3yearsold_), adult survival (*S*_adult_), 3-year-old fertility (*F*_3yearsold_), and adult fertility (*F*_adult_). Matrix transitions for (B) 2009–2010, (C) 2010–2011, (D) 2011–2012 and (E) 2012–2013.

We were interested in variables explaining variation in survival and reproduction, and if having successfully produced, a foal had any subsequent relationships with predicted survival. We initially constructed generalized linear mixed models (GLMMs) to test the combined effects of weather during the period of survival (*t* to *t *+* *1) and density (*t*) on survival for all age classes and sex. As climatic variables were highly correlated, we applied a PCA analysis (R package ade4) to compile our data into a common variable. We used the first axis of the PCA, which accounted for 90% of the variability of climatic variables, and faced hard winters (with high number of days below zero degrees) with mild winters and early springs (high mean temperature in January and high number of day above seven degrees). All models included individual identification number of horses as a random effect. Second, for 3-year-old and adult females, we constructed GLMMs to describe effects of density and climate on fecundity (presence/absence of a foal in year *t*). We included age as a two-level factor (3-year-olds or adult) and covariates of density and climate during the period of gestation (Fig.3).

We compared GLMMs using AIC corrected for small sample size (i.e., AIC_*c*_; Burnham and Anderson 2002). We selected the model with the smallest AIC_*c*_ including up to 2-way interactions; however, when the difference in AIC_*c*_ was <2.0 units between competing models, we applied the parsimony principle and selected the model with the smallest number of parameters (Burnham and Anderson 2002). We tested whether the assumptions for running generalized linear mixed models were met following Zuur et al. (2009). We performed all statistics using R, version 2.15.1 (R Development Core Team 2010).

## Results

**Figure 4:**
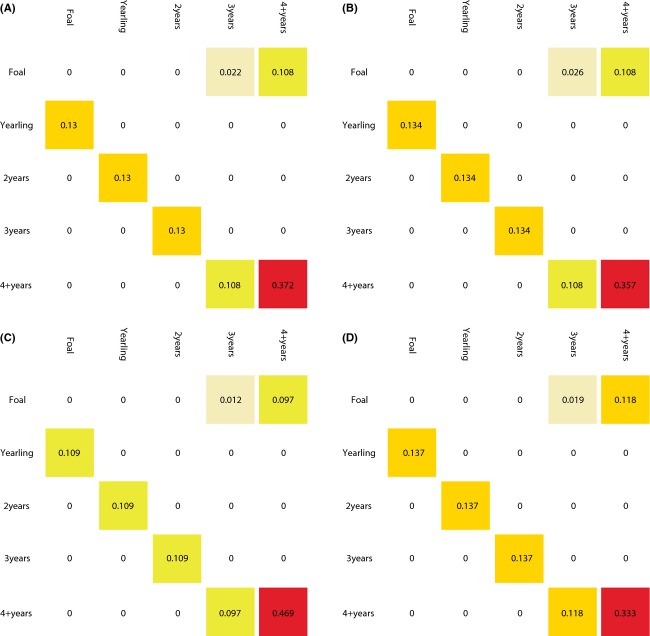
Values of elasticity rates of survival and reproduction (Fig.2) for Sable Island horses. (A) 2009–2010, (B) 2010–2011, (C) 2011–2012, and (D) 2012–2013.

**Figure 5:**
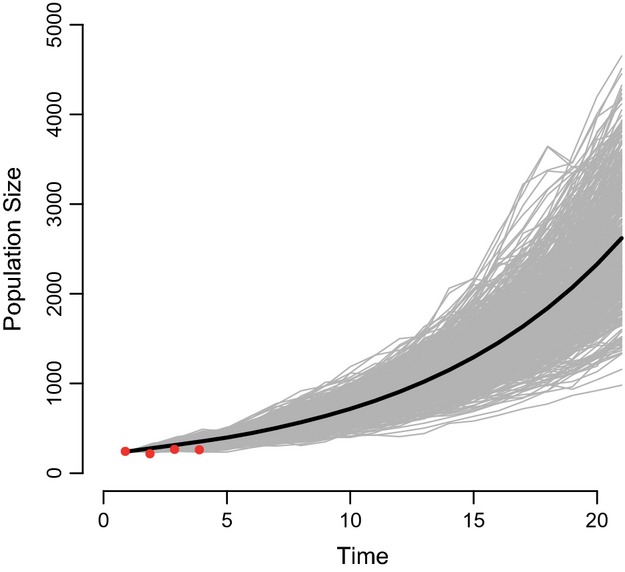
Population projection without density dependence for Sable Island horses on 20 years from observed values obtained during 4 years (2009–2013). Red points are observed values; the black line is the average increasing of the population; gray lines show variation in the size due to demographic and environmental stochasticity (included in SE of parameter estimates).

The total population increased at an annualized, finite rate of increase (*λ*) of 1.053 during the period of study (census years 2009–2013), with the only decline period occurring in 2010–2011 (*λ *= 0.909) and with reduced growth compared to other years in 2012–2013 (*λ *= 1.039). Annual survival of adult females (0.866 ± 0.107 [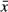 ± SE]) was lower than that of 3-year-olds (0.955 ± 0.051), although fecundity was higher in adults (0.616 ± 0.023) compared to 3-year-old horses (0.402 ± 0.054); the same pattern held for rates of reproduction, which included survival of foals to *t *+* *1 (0.508 ± 0.092 [adults], 0.345 ± 0.099 [3-year-olds]; estimates of fecundity and reproduction include births of both sexes). Two-year-old females, which showed no fecundity, survived at a mean rate that was intermediate between adults and 3-year-olds (0.888 ± 0.147). Foals and yearlings survived at rates of 0.876 ± 0.125 and 0.853 ± 0.078, respectively see Figure 3. Modeled changes in female adult survival rate had the most important consequence on population growth (greatest elasticity; Fig.4, females modeled only). After 20 years of projection (Fig.5), age structure shifted toward including less adults and more foals. The population at the equilibrium (stable age distribution) included 21.3% foals; 15.5% yearlings; 12.9% 2-year-olds; 10.5% 3-year-olds; and 39.7% adults, compared to an average of 16.8%; 15.0%; 10.8%; 9.1%; and 48.2%, respectively, from 2009 to 2013.

Survival was best modeled as a function of age, sex, density, and climatic conditions during the winter of survival, with interactions between age and sex, age and density, and density and climate (Table2; ranking of competing models in Supporting Information, Table S1). Adult male survival was less than that of females, except at high density. Density generally acted to reduce survival for all age categories, as did increasing severity of winters, although density interacted with climate. For reproducing females, survival was driven mainly by climatic conditions during the year of survival and density at the start of the survival period (Table2). When climate was mild, density had a negative effect on adult female survival; however, this reversed when winters were harsh. The selected model for fecundity included an effect of age, density at gestation, and climatic conditions during gestation (Table3; ranking of competing models in Supporting Information, Table S2). Females foaled at higher rates with increasing age; however, regardless of age, foaling rates decreased when winters were harsh and densities increased.

**Table 2:** Estimates and their significance for the selected generalized linear (binomial) mixed model predicting annual survival of Sable Island horses, 2009–2013. Categorical variables include sex (male = 1, female = 0), age (foal, yearling, 2-year old, 3-year old, and adult = 0), and continuous variables of density (horses/km^2^ at year *t*–1, i.e., at census prior to winter) and Climate (PCA scores on first axis for the winter of survival). The random effect was Horse ID with intercept 8.823^−17^ and SD 9.9393^−09^. Number of observations = 1961 from 701 horses. Deviance 1273.9; null deviance 1385.1

Variable	Estimate	SE	*z*	*P*
Intercept	10.709	2.300	4.656	<0.0001
Sex	*−*5.230	2.344	*−*2.231	0.026
Age (foal)	*−*0.851	2.945	*−*0.289	0.773
Age (yearling)	*−*5.062	3.390	*−*1.493	0.135
Age (2-year old)	11.341	5.683	1.995	0.046
Age (3-year old)	*−*1.325	6.028	*−*0.22	0.826
Density	*−*0.273	0.067	*−*4.104	<0.0001
Climate	6.139	1.513	4.058	<0.0001
Sex × Density	0.163	0.069	2.375	0.018
Age (foal) × Density	0.017	0.086	0.201	0.841
Age (yearling) × Density	0.155	0.101	1.528	0.127
Age 2-year-old) × Density	*−*0.325	0.163	*−*1.999	0.046
Age (3-year-old) × Density	0.075	0.178	0.423	0.672
Density × Climate	*−*0.180	0.042	*−*4.24	<0.0001

**Table 3:** Estimates and their significance for the selected generalized linear (binomial) mixed model predicting annual parturition of female Sable Island horses, 2009–2013. Variables included age (3-year-old = 1, adult = 0) and continuous variables of (horses/km^2^ at year *t*–1, i.e., at census prior to the onset of winter and during gestation) and climate (PCA scores on first axis during gestation). The random effect was Horse ID with intercept 0.02574 and SD 0.1604. Number of observations = 533 from 189 individual horses. Deviance 699.70; null deviance 723.96

Variable	Estimate	SE	*z*	*P*
(Intercept)	5.634	1.559	3.613	0.0003
Age	*−*0.885	0.250	*−*3.541	0.0004
Climate	0.249	0.094	2.643	0.0082
Density	*−*0.168	0.051	*−*3.321	0.0009

## Discussion

Climate and density can lead to important intercohort variations and have long-term effects on life history traits in ungulates (Forchhammer et al. 2001). Both of our environmental variables govern the availability of energy to Sable Island horses. Winter weather mediates the date of spring flush, quality of terrestrial forage during the winter, and accessibility of aquatic forage during the winter. High densities reduce the availability of forage through increased competition. Our results suggest that for female Sable Island horses, high density and poor weather during the period of gestation reduced fecundity; but, although density generally had a negative effect on survival, the same conditions also resulted in increased survival in adults.

Individuals of different age categories responded differently to variations in density, but responded similarly to variations in climatic conditions. Our results are partly in accordance with others studies of wild ungulate populations (Gaillard et al. 1998, 2000; Albon et al. 2000; Garrott et al. 2003), where prime-aged female survival is less variable between years compared to juvenile survival. In most populations, juvenile survival varies with environmental stochasticity, whereas adult survival remains constant (Gaillard et al. 1998, 2000). Here, we observed that juveniles (foals, yearlings, 2-year-olds) survived at lower rates than 3-year-olds and were more sensitive to variation in density; however, both foal and yearling survival were higher than that reported in other studies of ungulates (see Gaillard et al. 2000 for a review), although lower than that reported in another study of feral horses in Argentina (Scorolli and Cazorla 2010, *S*_foal_ = 0.94 and *S*_yearling_ = 0.87). Our observations are also in agreement with other studies showing density dependence in juvenile survival among several ungulate species (Clutton-Brock et al. 1987; Portier et al. 1998). As there is no predation on juveniles in our study area, we assume that foal survival strongly depends on maternal care and maternal attributes, as reported elsewhere (Festa-Bianchet et al. 1995 for maternal age; Cameron et al. 1993 for maternal body weight). In our population, maternal care should also affect yearling survival as mares continue to nurse until the next foal is born. The higher sensitivity of 2-year-olds (but not yearlings) to density points to maternal care for yearlings being a factor.

Adult survival was lower than both yearlings and 2-year-olds and was affected by an increase in density as reported for female horses by Grange et al. (2009). Across most ungulate populations, adult survival consistently ranks higher in elasticity than all other age-specific rates (e.g., Garrott et al. 2003 for elk, Johnson et al. 2010 for Sierra Nevada bighorn sheep, *Ovis canadensis sierrae*), but tends to show less variation when compared to juvenile survival, which is more vulnerable to limiting factors (Gaillard et al. 1998, 2000; Albon et al. 2000; Raithel et al. 2007). Indeed, adult females are phenotypically above average individuals because of the strong selection pressure (better individuals reach adult stage), and so, female survival should be more resistant to environmental variations and have little impact on population growth (trade-off between elasticity and temporal variation, see Gaillard et al. 2000). Female adult survival was lower in our study than was observed in other populations of feral horses [for instance: 0.95 for feral horses in the Great Basin, Berger (1986); 0.94 in Kaimanawa, Linklater et al. (2004)]; however, these values come from heavily managed populations and female adult survival decreased to 0.79 in the Camargue population when resources became limited (Grange et al. 2009). Low female adult survival may be explained by sustained increases in population size not only during the period of study, but likely for several years previous. We note that our current age distribution is not far removed from the projected stable age distribution, and hence, we can conclude that the population has been growing at a relatively constant rate for some time. The difference is that we see fewer foals compared to that at stable age distribution and greater numbers of adults compared to 20 years after projection. Typically, when a population increases, its age structure changes (Gaillard et al. 1998, 2000) leading to an increase in the average age of adults females and so greater adult mortality (Festa-Bianchet et al. 2003). Models of our population show 15% of the females and 11% of the males over 9 years of age. Increasing population density should have led to high juvenile mortality and lower fecundity, as observed in previous studies on ungulates (Gaillard et al. 1998, 2000) and then to a lower proportion of juveniles in the population in comparison with adults. That is not the case here or in other feral horse populations. Excluding foals, 39–50% of the Sable Island population was aged 1–3 years of age; this is similar to the stable age structure predicted by Garrott et al. (1991) for horses in the western United States. Moreover, this age structure is not linked with specific years as we obtained the same result when we projected the Leslie matrix over 20 years.

Unusually high overall adult female mortality rates may be explained by early onset of senescence. Senescence is expected for large herbivores (Albon et al. 2000 for red deer; Garrott et al. 2003 for elk); in feral horses, senescence has been estimated to occur after 15 years of age (Garrott and Taylor 1990). The population of horses on Sable Island is free from predators and hunting, so we are confident that senescence should occur; however, we require additional longitudinal data to study senescence. Whatever the explanation, our results may express some remaining evidence of past domestication (notwithstanding contrasts with the study of Grange et al. 2009).

Density negatively impacted survival for all age and sex categories; however, this appears to be mitigated by a harsh winter. Effects of reduced parturition on females is one explanation, but finding the interaction for all age and sex strata also suggests to us that social dynamics may play a role in this finding. Huddling behavior could be costly as it increases numbers of individuals on a small area, leading to competition for resources (Vickery and Millar 1984). However, if the gain to huddle counterbalances the cost, such behavior could lead to higher survival for both sex and age categories, depending on resources available and population density. Several species have been observed to huddle with their conspecifics in order to mitigate environmental (Helle et al. 1992; Satinoff 2011). Welsh (1975) mentioned that membership in large groups may benefit Sable Island horses during winter storms when huddling can reduce heat loss. Welsh (1975) also showed negative consequences on foal over-winter survival for individuals belonging to a small band at low density. A recent study in Barbary macaques (*Macaca sylvanus*) demonstrated for the first time that sociality can affect fitness in response to harsh winters (McFarland and Majolo 2013). Further research recording horse activity budgets and horse behavior may enlighten the importance of social behavior in population dynamics of horses and other large mammals.

Winter conditions did not influence foal survival any more than they did for other age categories; however, weather conditions during gestation can affect preweaning survival (Gaillard et al. 2000) and could explain the decreases of reproduction in both 3-year-old and adult horses when the severity of winter increases. Similarly, adverse weather conditions can lead to low fecundity of young females and population density can affect age at primiparity (Kie and White 1985). During all years of the study, reproduction of young females was lower than reproduction of adult females, but the response to environment or density was the same. This suggests that environmental conditions and resource acquisition\-mediated reproduction in the same way for both 3-year-olds and adults, and that lower reproduction in younger females may be explained by variations in age at primiparity depending on cohort effects.

Our finding that adult females did not maintain high reproductive performance at high density, and their survival did not necessarily decrease, is at odds with the hypothesis of Grange et al. (2009) that prior artificial selection for high reproductive performance might decouple the expected trade-off in survival and reproduction in feral horses. This was the case for Camargue horses, which agreed with other studies suggesting that feral ungulates including cattle and sheep respond differently from wild ungulates to increases in density, by trading adult survival for reproduction. Finding that Sable Island horses responded as expected for wild ungulates in general, and not like that of Camargue horses, suggests fundamental differences in the life history of two populations of the same species. In contrast to ruminants, which have been selected principally for their milk or meat production (Koenen et al. 2004), horses (especially saddle horses) have been selected on the basis of other criteria, including athletic or working ability (Clutton-Brock 1981). Such selection could influence variations in life history traits. These differences may lie not only in historical differences regarding the course of artificial selection, but more importantly the period of feralization.

Our study increases our knowledge of the combined effects of density and weather on both survival and reproduction for a large, long-lived herbivore. It is unique in the sense that we present a very detailed analysis of the dynamics of an isolated population with a history of artificial selection, long period of feralization, rapid growth with clear effects of density dependence, under conditions of no predation, interspecific competition, or human interference. Our results inform us as to the life history consequences that we can expect for large ungulates under these controlled conditions.

## Supporting Information

**Table S1.** Model selection for the analysis of variation in annual probability of survival for Sable Island horses, 2009–2013 (top ten models only). **Table S2.** Model selection for the analysis of variation in annual fecundity (probability of presenting with a foal) for Sable Island horses, 2009–2013 (top ten models only).
