# Supplementary material for: Interacting effects of age, density, and weather on survival and current reproduction for a large mammal
Source: Ecol Evol. 2014 Sep 18;4(19):3851–60. doi: 10.1002/ece3.1250 (PMC4301048; doi:10.1002/ece3.1250)
Supplement: Supplementary file 1 — Table S1. Model selection for the analysis of variation in annual probability of survival for Sable Island horses, 2009–2013 (top ten models only). Table S2. Model selection for the analysis of variation in annual fecundity (probability of presenting with a foal) for Sable Island horses, 2009–2013 (top ten models only). [file ece30004-3851-sd1.docx]

**Supplementary Information, Richard et al.**

**Table S1.** Model selection for the analysis of variation in annual probability of survival for Sable Island horses, 2009–2013 (top ten models only). The table displays the fit of the effects of sex (2-level factor [reference is female]), age (5-level factor [reference is adult]), first axis of the PCA that resumes climatic variables during the period of survival (Climate) and conspecific density as measured prior to the period of survival. All models include individual identity as a random effect. Models are ranked according to their AIC*_c_*. Selected model (smallest AIC*_c_* or most parsimonious when difference of AIC*_c_* is less than 2.0) is in bold (df = degrees of freedom, logLik = log likelihood, w = model weight). Null model AIC*_c_* = 1385.1).

| Model | Included variables | | | | | | | | | | df | logLik | AIC*_c_* | ΔAIC*_c_* | *w* |
| --- | --- | --- | --- | --- | --- | --- | --- | --- | --- | --- | --- | --- | --- | --- | --- |
| 1 | Age | Climate | Density | Sex | Age × Climate | Age × Density | Age × Sex | Climate × Density | | Sex × Density | 23 | -628.07 | 1302.7 | 0.0 | 0.36 |
| 2 | Age | Climate | Density | Sex | Age × Climate | Age × Density | | Climate × Density | | Sex × Density | 19 | -632.27 | 1302.9 | 0.2 | 0.29 |
| **3** | **Age** | **Climate** | **Density** | **Sex** |  | **Age × Density** | | **Climate × Density** | | **Sex × Density** | **15** | **-636.94** | **1304.1** | **1.4** | **0.09** |
| 4 | Age | Climate | Density | Sex |  | Age × Density | Age × Sex | Climate × Density | | Sex × Density | 19 | -632.89 | 1304.2 | 1.5 | 0.08 |
| 5 | Age | Climate | Density | Sex | Age × Climate | Age × Density | Age × Sex | Climate × Density | Climate × Sex | Sex × Density | 24 | -627.99 | 1304.6 | 1.9 | 0.05 |
| 6 | Age | Climate | Density | Sex | Age × Climate | Age × Density | | Climate × Density | Climate × Sex | Sex × Density | 20 | -632.21 | 1304.8 | 2.1 | 0.04 |
| 7 | Age | Climate | Density | Sex |  |  |  | Climate × Density | | Sex × Density | 11 | -641.50 | 1305.1 | 2.4 | 0.03 |
| 8 | Age | Climate | Density | Sex |  | Age × Density | | Climate × Density | Climate × Sex | Sex × Density | 16 | -636.84 | 1306.0 | 3.3 | 0.01 |
| 9 | Age | Climate | Density | Sex |  | Age × Density | Age × Sex | Climate × Density | Climate × Sex | Sex × Density | 20 | -632.83 | 1306.1 | 3.4 | 0.01 |
| 10 | Age | Climate | Density | Sex |  |  | Age × Sex | Climate × Density | | Sex × Density | 15 | -637.97 | 1306.2 | 3.5 | 0.01 |

**Table S2.** Model selection for the analysis of variation in annual fecundity (probability of presenting with a foal) for Sable Island horses, 2009–2013 (top ten models only). The table displays the fit of the effects age (2-level factor [reference is Adult]), first axis of the PCA that resume climatic variables during the period of gestation (Climate) and conspecific density as measured during gestation. All models include individual identity as a random effect. Models are ranked according to their AIC*_c_*. Selected model (smallest AIC*_c_* or most parsimonious when difference of AIC*_c_* is less than 2.0) is in bold (df = degrees of freedom, logLik = log likelihood, w = model weight). Null model AIC*_c_* = 725.96.

| Model | Included variables | | | | | | df | logLik | AIC*_c_* | ΔAIC*_c_* | *w* |
| --- | --- | --- | --- | --- | --- | --- | --- | --- | --- | --- | --- |
| **1** | **Age** | **Climate** | **Density** |  |  |  | **5** | **-349.86** | **709.8** | **0.00** | **0.23** |
| 2 | Age | Climate | Density | Age × Climate |  |  | 6 | -348.86 | 709.9 | 0.04 | 0.23 |
| 3 | Age | Climate | Density |  | Age × Density | | 6 | -349.33 | 710.8 | 0.98 | 0.14 |
| 4 | Age | Climate | Density | Age × Climate | Age × Density | | 7 | -348.83 | 711.9 | 2.05 | 0.08 |
| 5 | Age | Climate | Density |  |  | Density × Climate | 6 | -349.86 | 711.9 | 2.05 | 0.08 |
| 6 | Age | Climate | Density | Age × Climate |  | Density × Climate | 7 | -348.86 | 711.9 | 2.10 | 0.08 |
| 7 | Age | Climate | Density |  | Age × Density | Density × Climate | 7 | -349.33 | 712.9 | 3.03 | 0.05 |
| 8 | Age | Climate | Density | Age × Climate | Age × Density | Density × Climate | 8 | -348.83 | 713.9 | 4.11 | 0.03 |
| 9 | Age | Climate | Density | Age × Climate | Age × Density | Density × Climate | 9 | -347.95 | 714.2 | 4.40 | 0.03 |
| 10 | Age |  | Density |  |  |  | 4 | -353.44 | 715.0 | 5.12 | 0.02 |
